# Development of transferability guidance for integrated care models with special focus on Central and Eastern European countries

**DOI:** 10.3325/cmj.2020.61.252

**Published:** 2020-06

**Authors:** Zoltán Kaló, Antal Zemplényi, Maureen Rutten-Van Molken, Willemijn Looman, Mirjana Huić, Romana Tandara, Haček, Guenka Petrova, Oresta Piniazhko, Tomas Tesar, Marcell Csanádi, János G. Pitter

## Abstract

**Aim:**

To develop pragmatic recommendations for Central and Eastern European (CEE) policymakers about transferability assessment of integrated care models established in higher income European Union (EU) countries.

**Methods:**

Draft recommendations were developed based on Horizon 2020-funded SELFIE project deliverables related to 17 promising integrated care models for multimorbid patients throughout Europe, as well as on an online survey among CEE stakeholders on the relevance of implementation barriers. Draft recommendations were discussed at the SELFIE transferability workshop and finalized together with 22 experts from 12 CEE countries.

**Results:**

Thirteen transferability recommendations are provided in three areas. Feasibility of local implementation covers the identification and prioritization of implementation barriers and proposals for potential solutions. Performance measurement of potentially transferable models focuses on the selection of models with proven benefits and assurance of performance monitoring. Transferability of financing methods for integrated care explores the relevance of financing methodologies and planning of adequate initial and long-term financing.

**Conclusions:**

Implementation of international integrated care models cannot be recommended without evidence on its local feasibility or scientifically sound and locally relevant performance assessment in the country of origin. However, if the original financing method is not transferable to the target region, development of a locally relevant alternative financing method can be considered.

In Central and Eastern Europe (CEE), life expectancy at birth is five to ten years shorter than in Western European countries (1). Inequalities in multimorbidity between Western and Eastern parts of Europe are hard to quantify due to the differences in multimorbidity definitions and data collection methods between national studies. Nevertheless, in a multinational cross-sectional analysis of the 50+ EU population in the Survey on Health, Aging and Retirement in Europe, the three countries with highest self-reported, age-adjusted prevalence of multimorbidity were Hungary, Estonia, and Poland (2). When large EU regions were compared, the highest self-reported, age- and sex-adjusted prevalence of multimorbidity was found in CEE (35.2%), followed by Western (34.8%), Southern (29.8%), and Northern Member States (26.2%) (3).

As a response to aging societies and the increasing prevalence of multimorbidity throughout Europe, integrated care has been receiving more attention, and several integrated care initiatives have been developed and implemented for better care of patients with multimorbidity (4-6). However, there are very few integrated care models in former socialist EU Member States (7). In the recently completed EU funded ICARE4EU project, country experts from 25 EU countries identified 101 corresponding practices or programs. However, in the CEE region very few models were found, and no relevant models were identified in the Czech Republic, Hungary, Poland, Slovakia, and Romania (8).

Based on the greater health burden and the lower development level of health systems, the CEE region has most room for health system improvement in the EU. Still, this region receives disproportionally low health-related research and innovation support from EU sources. Although one in five EU citizens live in a CEE country, CEE countries received only 3% of health-related FP7 and H2020 research grants in the 2007-2016 period. On average, beneficiaries from the CEE region received half of the budget of their EU-15 peers, had a lower chance to get involved in a second project, and a very low chance to act as project coordinator (9). Accordingly, knowledge transfer from more matured health systems and health policy-related research projects to the CEE region should be increasingly encouraged and facilitated.

In this study, transferability refers to the extent to which an integrated care model that has been already established in a country can be implemented in the specific context of another country with similar added value as in the country of origin. As more affluent Western European countries with healthier populations receive significantly more EU funding for health care research compared with CEE countries (9), transferability assessment of EU funded research project deliverables is more important from the perspective of lower income than higher income European countries. Hence, our aim was to provide pragmatic recommendations for policymakers in CEE countries about the transferability assessment of integrated care models already established in other countries.

The work presented in this article was completed within the SELFIE H2020 project. The SELFIE project (Sustainable integrated chronic care modeLs for multi-morbidity: delivery, FInancing, and performancE) was a Horizon2020-funded EU project that aimed to contribute to the improvement of person-centered care for persons with multimorbidity by proposing evidence-based, economically sustainable, integrated care programs that stimulate cooperation across health and social care and are supported by appropriate financing and payment schemes. Specifically, the project aimed to develop a taxonomy of promising integrated care programs for persons with multimorbidity; provide evidence-based advice on matching financing/payment schemes with adequate incentives to implement integrated care; provide empirical evidence on the impact of promising integrated care on a wide range of outcomes using Multi-Criteria Decision Analysis; develop implementation and change strategies tailored to different care settings and contexts in Europe, especially CEE (10,11).

The SELFIE consortium includes eight organizations in the following countries: the Netherlands (coordinator), Austria, Croatia, Germany, Hungary, Norway, Spain, and the UK (*www.selfie2020.eu*) [Grant Agreement No 634288].

The project had four components that together ensured a comprehensive approach to transferability assessment. The first key component was reasonable economic diversity of countries in the SELFIE consortium, involving research partners also from the CEE region (Croatia and Hungary). As opposed to many other EU funded research projects, CEE participants had a substantial role in the entire project. The second key component was that CEE consortium members had the opportunity to actively contribute to key work packages of SELFIE and consider transferability aspects upfront in all activities throughout the project. Third, the project scrutinized four integrated care models also from the CEE region to test whether the same sophisticated methodology as for 13 models from EU-15 countries would be also applicable in CEE countries. And last but not least, a stand-alone work package was dedicated to the transferability of project deliverables to CEE countries under the leadership of CEE institutes.

## MATERIAL AND METHODS

### Development of draft transferability guidance

As a first step of the guidance development, a multi-stakeholder survey was conducted to identify key barriers to integrated care implementation in CEE (12). Eighty-one relevant stakeholders from Croatia, Hungary, Poland, Romania, and Serbia revealed that the most critical implementation barriers were long-term financial sustainability and lack of dedicated financing schemes, followed by the lack of integration between health and social care providers and insufficient availability of human resources.

The draft transferability guidance was composed by the CEE consortium members of the SELFIE project based on a thick description of 17 promising integrated care models for multimorbid patients throughout Europe – with special emphasis on the four integrated care models focusing on oncology care, palliative care, and care for the elderly in Hungary and Croatia – and the survey results about implementation barriers in CEE. The draft guidance was refined in iterative sessions by considering certain requirements, such as completeness, non-redundancy, non-overlap, and preference independence of recommendations. To provide sufficient details but to preserve clarity, each recommendation had a short form and a detailed description.

### The transferability workshop and finalization of transferability guidance

As a final step, a face-to-face one-day transferability workshop was organized in Zagreb in June 2019 to review, amend, and validate the draft recommendations. In order to make sure that the workshop participants have sufficient time to express and discuss their own opinions, only a highly selected expert group representing multiple stakeholders from 12 CEE countries was invited. Workshop participants were identified in an iterative process by exploiting the professional networks of SELFIE partners. The selection criteria were familiarity with health systems and familiarity with the needs of patients with chronic multimorbidity and integrated care models in CEE. Efforts were made to achieve a balanced distribution of participants regarding their country of residence and primary stakeholder perspective. Of the 30 invited experts from Eastern Europe, 22 accepted the invitation, including 6 stakeholders from Croatia, 3 from Hungary, 2 from Poland and Romania, and 1 from Bosnia and Herzegovina, Bulgaria, Czech Republic, Estonia, Serbia, Slovenia, Slovakia, and Ukraine. Eight experts represented academic research institutes, 4 policy-making bodies, 3 third-party payers, 5 patient organizations, and 2 informal caregivers. There were 10 researchers from 5 SELFIE partners, who also actively supported the workshop by moderating sessions, making presentations, answering the invited experts’ questions, and writing minutes.

The discussion started with introductory presentations on the SELFIE multimorbidity integrated care framework, methods of qualitative analysis and performance assessment of the selected models, the developed multi-criteria decision analysis tool, lessons learned in EU-15 countries about various financing methods with incentives for integrated care, and the CEE stakeholder survey findings on perceived key barriers to integrated care models in the CEE region that were identified by the SELFIE project.

Following the introduction, the draft guidance was presented and discussed in three consecutive steps, including feasibility of transferring programs, transferability of international performance assessment, and local adaptation of financing and payment methods. The discussion was moderated by the first author of this article and facilitated by the brief presentation of the corresponding details of a selected case study, a project called Learning Networks, implemented in Norway (13). Case study selection was based on high local relevance in CEE, according to the findings of the SELFIE project, and on the availability of completed Multi-Criteria Decision Analysis report at the time of workshop preparation. Participants were asked to share their recommendations to a hypothetical CEE country either in generalized format or specifically referring to the transferability of the presented case study. Stakeholder feedback that specifically addressed the specific case study was immediately abstracted and translated by the moderator to not case-specific, general recommendations. The generalized content was directly cross-checked and verbally confirmed by all workshop participants as part of the discussion. The discussion was continued until consensus on each recommendation was reached.

After the workshop, the draft guidance was reviewed by SELFIE partners based on the workshop minutes and post-workshop written correspondence with the participants. The final guidance was released to SELFIE partners involved in the guidance development and workshop participants for their final comments and approval. All workshop participants agreed with the final transferability guidance.

## RESULTS

The transferability guidance provides 13 practical recommendations in three major areas: (I) the feasibility of transferring evidence-based integrated care models, (II) performance measurement of potentially transferable integrated care models, and (III) transferability of financing methods for integrated care models (Table 1).

**Table 1:** The structure of transferability recommendations related to the integrated care models

Major areas	Summary of recommendations
**(I) The feasibility of transferring evidence-based integrated care models**	1. Identify potential barriers to implementing integrated care models
	2. Prioritize the identified barriers according to their local relevance
	3. Generate consensus among multiple local stakeholders on local feasibility and on potential solutions to key barriers
	4. Publish findings to extend the evidence base of integrated care models
**(II) Performance measurement of potentially transferable integrated care models**	5. Select evidence-based integrated care models
	6. Proven benefits should be relevant in the local context
	7. Estimate the magnitude of potential benefits in the local context
	8. Policy-relevant methodology should be applied for aggregation of performance in multiple domains
	9. Consistent and transparent decision rule should be applied
	10. Continuous performance monitoring of implemented integrated care models is needed
**(III) Transferability of financing methods for integrated care models**	11. Locally relevant financing methodology is needed
	12. All aspects of health care financing should be considered
	13. Plan for adequate initial and long-term financing

### Feasibility of transferring evidence-based integrated care models

Recommendation 1: Identify potential barriers to implementing integrated care models

Identification of the main barriers and facilitators is necessary to address the feasibility of transferring integrated care models. An optimal methodology for retrieving a wide selection of known barriers and facilitators is the review of the scientific literature (14,15) and description of relevant integrated care models. Taxonomy of key barriers should be created by categorization of individual barriers and facilitators (eg, leadership and governance, service delivery, workforce, financing, technology and medical product, information and research).

Recommendation 2: Prioritize the identified barriers according to their local relevance

Local relevance of a wide range of known barriers is necessary to narrow the focus to the most important items in the target country or region. A survey among a wide range of multiple stakeholders about relative importance of barriers can improve the objectivity of prioritization.

Recommendation 3: Generate consensus among multiple local stakeholders on local feasibility and on potential solutions to key barriers

Workshops with deliberative process can help to identify potential solutions and create consensus recommendations for the most important barriers. Workshop participants should represent multiple stakeholders, including policymakers, payers, service providers, patients, and informal caregivers. This (or a similar) workshop should conclude with a decision on the feasibility of transferring the original model to local jurisdictions in the new target country or region. The feasibility assessment can be supported by recommendations on how to overcome the barriers and make most use of the facilitators.

Recommendation 4: Publish findings to extend the evidence base of integrated care models

The scientific literature on the evidence base of policy decisions related to the implementation of integrated care models is fairly limited, especially in lower income countries. International dissemination of transferability assessment can improve the methodological approach of evaluating integrated care models and their evidence base.

### Performance measurement of potentially transferable integrated care models

Recommendation 5: Select evidence based integrated care models

In lower income countries, scarce health care resources should not be spent on technologies or programs without a convincing evidence base. Transferring of an existing integrated care model should not be considered if the original program has no scientifically sound performance assessment. Even the most promising ideas and programs may fail, hence evidence-informed health policy necessitates the availability of positive findings in key outcome parameters in the country of origin.

Recommendation 6: Proven benefits should be relevant in the local context

Relevance and completeness of key outcome parameters should be evaluated from the local perspective of the new target country or region. If the most important outcome parameters (eg, patient survival) have not been measured in the country of origin, transferring the integrated care model cannot be based on scientifically sound performance assessment (see Recommendation 5).

Recommendation 7: Estimate the magnitude of potential benefits in the local context

There might be differences between the performance of the original and the transferred integrated care models for many reasons, including differences in target populations, usual care patterns, education and preparedness of model teams, and other health care system features. Transferability of each key outcome parameter should be assessed both qualitatively and quantitatively. Sensitivity analysis should be performed to understand the implications in the uncertainty of key outcome variables. In general, cost (or resource utilization) outcomes are the least transferable variables, because local production functions and unit costs are different among countries. Patient experience may also be more context- or country-specific than health outcomes.

Recommendation 8: Policy-relevant methodology should be applied for aggregation of performance in multiple domains

Aggregation of performance in multiple outcomes is necessary to assess the overall benefit of integrated care models. The starting point for aggregating key outcomes can be an existing method of making evidence-based decisions, as many CEE countries routinely perform cost per quality-adjusted life-year calculations with explicit thresholds. If multiple criteria decision analysis tool is acceptable for policy-makers to aggregate all relevant outcome parameters, it is necessary to determine the relative weights for each criterion. Often there is limited budget to conduct survey to elicit local weights, hence using scores from a “similar” country or eliciting weights in a policy workshop with limited budget is a better option than applying weights from the country of origin.

Recommendation 9: Consistent and transparent decision rule should be applied

Decision rule for aggregated performance assessment has to be established in the target country or region before evaluation of integrated care models. Even if the decision rule is applied initially for a single case, using a generalizable rule for future multiple integrated care models improves the consistency and transparency of policy decisions.

Recommendation 10: Continuous performance monitoring of implemented integrated care models is needed

Monitoring the performance of the implemented integrated care model is necessary to understand how outcomes in the new target country or region deviate from the outcomes in the country of origin. If local performance is below the expected performance level, adjustment or even termination of the transferred model should be considered.

### Transferability of financing methods for integrated care models

Recommendation 11: Locally relevant financing methodology is needed

Transferability of financing methods is not a mandatory condition for transferring integrated care models from other jurisdictions. However, as a first step it is highly recommended to explore whether the financing method in the country of origin could be adaptable for local implementation. If the financing method is not transferable and cannot be adapted, a financing methodology should be developed for the target country or region.

Recommendation 12: All aspects of health care financing should be considered

Development or adaptation of financing methods should ensure all important aspects of health care financing, including a) fund raising, b) allocation of resources, and c) financial incentives for all health and social care providers in the new target country or region.

Recommendation 13: Plan for adequate initial and long-term financing

Necessary resources should be available for the initial implementation period (covering also setup costs) and long-term operation of the integrated care model.

## DISCUSSION

Our study provides 13 practical recommendations for the transferability assessment of integrated care models. The guidance contains some recommendations that specifically address the problems of CEE countries, while some other recommendations are not specific but highly relevant also in these countries. The specific focus on CEE is required because these countries have poorer health status and more limited health care resources compared with Western Europe, and consequently have a greater opportunity cost of inappropriate policy decisions. Chronic patients with multimorbidity necessitate integrated care models, however, in CEE countries it is not cost-effective to develop models from scratch if existing international models with proven benefits can be adapted. Therefore, in this article we advocate the local adaptation of international integrated care models based on a thorough evaluation of the best available evidence. One could argue that a technology or program with limited added value in Western Europe could still have more significant added value in CEE countries, since it is possible that in the original country there was little room for improvement due to the high baseline level of care. However, given the wide range of existing integrated care models in Western Europe and limited tradition for transparent and evidence-based health policy decisions in CEE countries (16), only evidence-based models should be preferred when selecting a candidate for transferability assessment to less developed regions.

Current health policy articles on transferability mainly focus on economic evaluations of health technologies (17-20). The majority of publications refer to the original concept of Welte et al (21) related to the transferability of economic evaluations and categorize different domains of scientific evidence as not transferable (ie, general knockout criterion), transferable after adjustment (ie, specific knockout criterion), and transferable without adjustment. However, transferability assessment should be extended to assessing the feasibility of implementing valuable health care services and technologies in countries with different patient populations and health care systems. Transferability of evaluation methods to support the evidence base of policy decisions also needs to be carefully assessed due to cultural differences and political context in decision-making processes.

In our guidance we applied this broader policy context for the transferability assessment of integrated care models. The proposed three-step assessment should include the feasibility assessment of transferring international integrated care models, followed by the transferability of performance assessment, and finally the evaluation of financing and payment methods.

If transferring an international integrated model is not feasible or if there is no scientifically sound and locally relevant performance assessment of the integrated model in the country of origin, local implementation of an international integrated care model cannot be recommended. These two main domains are considered as general knockout criteria. However, if the financing method in the country of origin is not transferable, development of a new financing method with appropriate fund raising, resource allocation, and financial incentives can be considered, hence the financing method should be considered as a specific knockout criterion. Nevertheless, a dedicated set of recommendations on financing aspects is justified by the assumption that the current payment mechanisms may not create appropriate incentives for providing integrated care (22) and by the high priority of financing barriers in a recently conducted transferability survey among CEE experts (12).

In our 13 recommendations we encourage countries to seek simplicity and transparency in all aspects of implementing international integrated care models. Tailored guidance may contribute to feasible, transparent, stepwise, and standardized health policies in CEE countries with restricted financial and human research capacities. It is our hope that applying these recommendations will lead to better care for patients with chronic multimorbidity, while improving the sustainability of health care financing. It should be noted that our transferability guidance is based primarily on empirical observations, a stakeholder survey, and the opinion of a relatively small-sized expert group, which is the most important limitation of our study. Hence, future research should test the appropriateness of our recommendations by conducting smaller-scale studies focusing on particular integrated care models. Our guidance should be viewed as a first step in a multi-stakeholder dialogue about practices of transferring integrated care models.
